# Climatic niche and potential distribution of *Tithonia diversifolia* (Hemsl.) A. Gray in Africa

**DOI:** 10.1371/journal.pone.0202421

**Published:** 2018-09-05

**Authors:** Maxwell C. Obiakara, Yoan Fourcade

**Affiliations:** 1 Department of Botany, Ecology Unit, University of Ibadan, Ibadan, Nigeria; 2 Department of Ecology, Swedish University of Agricultural Sciences, Uppsala, Sweden; Universita degli Studi di Napoli Federico II, ITALY

## Abstract

Mexican sunflower, *Tithonia diversifolia* (Asteraceae), is an invasive tropical plant species native to Central America. It has spread in more than 70 countries across Asia, Africa and Australia. In Africa, this species is known to disturb native crops and plant communities, but its negative impacts remain underestimated. Moreover, its potential invasion risk has not been investigated so far. A fundamental aspect in the identification and prediction of habitats susceptible to biological invasions lies in the ability of an organism to conserve or change its ecological niche as part of the invasion process. Here, we compared the realised climatic niche of *T*. *diversifolia* between its Central American and African ranges. In addition, reciprocal distribution models were calibrated on its native and invaded ranges. Models were combined and projected to current and future climatic conditions in Africa to estimate the potential distribution of this species. Niche overlap given by Schoner's D index was low (0.23), equivalency and similarity tests suggested that the climatic niche of *T*. *diversifolia* is not similar in both ranges. However the low expansion (*U* = 0.09) and very high stability (*S* = 0.92) indices support climatic niche conservatism for this species in Africa, although it has not filled its entire niche so far. Our combined reciprocal models highlight highly suitable areas for this species in humid regions throughout East, Central and West Africa, then in some parts of South Africa and Madagascar. Future projections indicated that the distribution of climatically suitable habitats will likely remain stable.

## Introduction

In recent decades, humans have either intentionally or accidentally introduced alien species into new areas at increasing rates [1–3]. This has resulted in about 3.9% of the global vascular flora forming self-sustaining populations in introduced regions over the globe [2]. Biological invasions mainly lead to local biodiversity losses [4,5], changes in nutrient cycling [6,7] and loss of ecosystem services in invaded areas [8–10]. Today, invasions by alien plant species constitute a pressing global issue with severe ecological and economic consequences. Furthermore, with increasing connections among biotically disparate regions of the world by means of fast movements of people and propagules, biological invasions are expected to be more frequent in the future [11]

Mexican sunflower, *Tithonia diversifolia* is an annual shrub native to several Central American countries including Mexico, Cuba, Honduras and Costa Rica [12]. This species has been introduced in more than 70 countries mainly as an ornamental plant and became invasive throughout tropical, subtropical and warm temperate regions of the world [13]. Its first known introduction in Africa was through Nigeria in the late 1970s [14]. Mexican sunflower is currently spreading rapidly throughout tropical and sub-tropical Africa and is already dominating several ecological regions. In Nigeria, this plant typically forms large, monospecific and impenetrable populations in open, sunlit habitats such as road verges and adjacent arable lands. *T*. *diversifolia* requires high temperatures and light intensities for optimal growth but does not tolerate water stress [15,16]. Traits that explain the high invasiveness of Mexican sunflower include rapid vegetative growth, prolific seed production [17,18] and allelopathic potential [19]. Although this species is of medicinal and ethnopharmacological importance in Africa [20], it has been known for its growth-inhibiting effects on a wide range of important crops [20,21].

In the last decades, there has been an increasing interest in understanding the distribution patterns of organisms at local and global scales amidst threats posed by invasive alien species. To explore the behaviour of introduced species in novel habitats, scientists have used models also referred to as species distribution models, ecological niche models or habitat suitability models [22]. These models establish the relationship between species presences and optionally absences at certain locations and abiotic conditions using mathematical functions [23]. By modelling species’ niche in the environmental space, then projecting it into geographical space, these approaches allow to build predictive maps of species’ potential distributions in the absence of dispersal or biotic constraints [24]. Because of the scarcity of information regarding local-scale drivers of species distributions, and because of the assumption that climate is the main factor determining range boundaries [25], distribution models have often relied on species’ climatic niche only. Climate niche models and species distribution models in general have been widely used in biodiversity assessments [26,27], conservation biology [28–30] and invasive species monitoring [31–34]. Invasive species distribution modelling focuses on risk assessments and develops risk maps for such species [31,32,35].

Approaches that aim at predicting species’ invasive potential are based on the assumption of niche conservatism, i.e. the propensity of invasive species to retain their ancestral niche in novel habitats [36]. Although niche conservatism has been observed in introduced plants [37], an increasing number of studies support prevalent niche shifts in invasive species[38–41]. Niche shifts can exhibit various patterns, niche unfilling occurs when a species occupies a reduced subset of its native ecological niche. This usually results from dispersal limitations and is expected to decrease during invasion as the species gradually breaks through its geographical barriers, spreads and fills its entire niche [42]. On the other hand, niche expansion is observed when a species occupies a range of climatic conditions where it is absent in its native range [42]. Niche expansion can be a result of evolution in a species fundamental niche through adaptation during the invasion process [43], or a shift in its realized niche if, for example, release from biotic constraints in the invaded range allows it to fill its entire fundamental niche [44]. In addition, a species may be pre-adapted to conditions that it did not experience in its native range, therefore showing a shift in its realised niche driven by the availability of these conditions in the adventive range [42]. Niche dynamics of introduced species present important implications for our ability to predict potential areas of invasions. Thus, there is a need to understand the incidence and importance of niche shifts in invasive plants.

This study evaluated the ongoing range expansion of *T*. *diversifolia* in Africa and the role of the climatic niche of this plant in its invasion success. We assessed the following questions: Has the climatic niche of *T*. *diversifolia* changed since its introduction in Africa? Has this species filled its native climatic niche in Central America? We also map the current and future potential distribution of *T*. *diversifolia* in order to provide baseline information necessary for its control.

## Materials and methods

### Study species

Mexican sunflower, *T*. *diversifolia* is a shallowly rooting monocarpic annual shrub that can grow up to six metres high. It has large alternate lobed leaves (up to 45 cm long) and bright yellow capitula. This plant usually forms impenetrable stands and has a pithy stem typically un-branched at high densities (8–20 plants/m^2^). In rare cases, *T*. *diversifolia* branches profusely and can reach up to 10 metres high when growing solitarily. This species spreads mainly by sexual reproduction and each individual can produce several thousand achenes [17]. *T*. *diversifolia* thrives in open, sunny and ruderal habitats, along river courses and road verges [45,46]. In Nigeria, Mexican sunflower germinates in April at the beginning of the rainy season, flowering starts in August and seeds are dispersed from November to January. Since its accidental introduction in this country, this plant has spread widely and is becoming a major weed of arable lands [46]. *T*. *diversifolia* produces large amounts of allelopathic compounds and rapidly attains dominance in invaded communities [19].

### Definition of the native and invaded ranges and occurrence records

*T*. *diversifolia* is native to Belize, Costa Rica, Guatemala, Honduras, Jamaica, Mexico, Nicaragua and Panama [12]. Although this plant has invaded several Asian countries including China, India, Nepal and Philippines, this study was constrained to Africa where a considerable number of occurrence records exist and its impacts are more pronounced [17,18,45,46,47]. Distributional records for this species were obtained from the Global Biodiversity Information Facility (www.gbif.org/) using the dismo R package [48]. After excluding ambiguous and erroneous occurrences (e.g. records listed outside the countries/regions defined above), spatial filtering was applied to records between 1960 and 2000 using the spThin R package [49]. A thinning distance of 5 km and 100 replicates resulted in 251 and 39 records in Central America and Africa respectively. This step has been shown to substantially improve model performance by reducing sampling bias that may arise when some areas are disproportionally surveyed [50,51].

**Fig 1:**
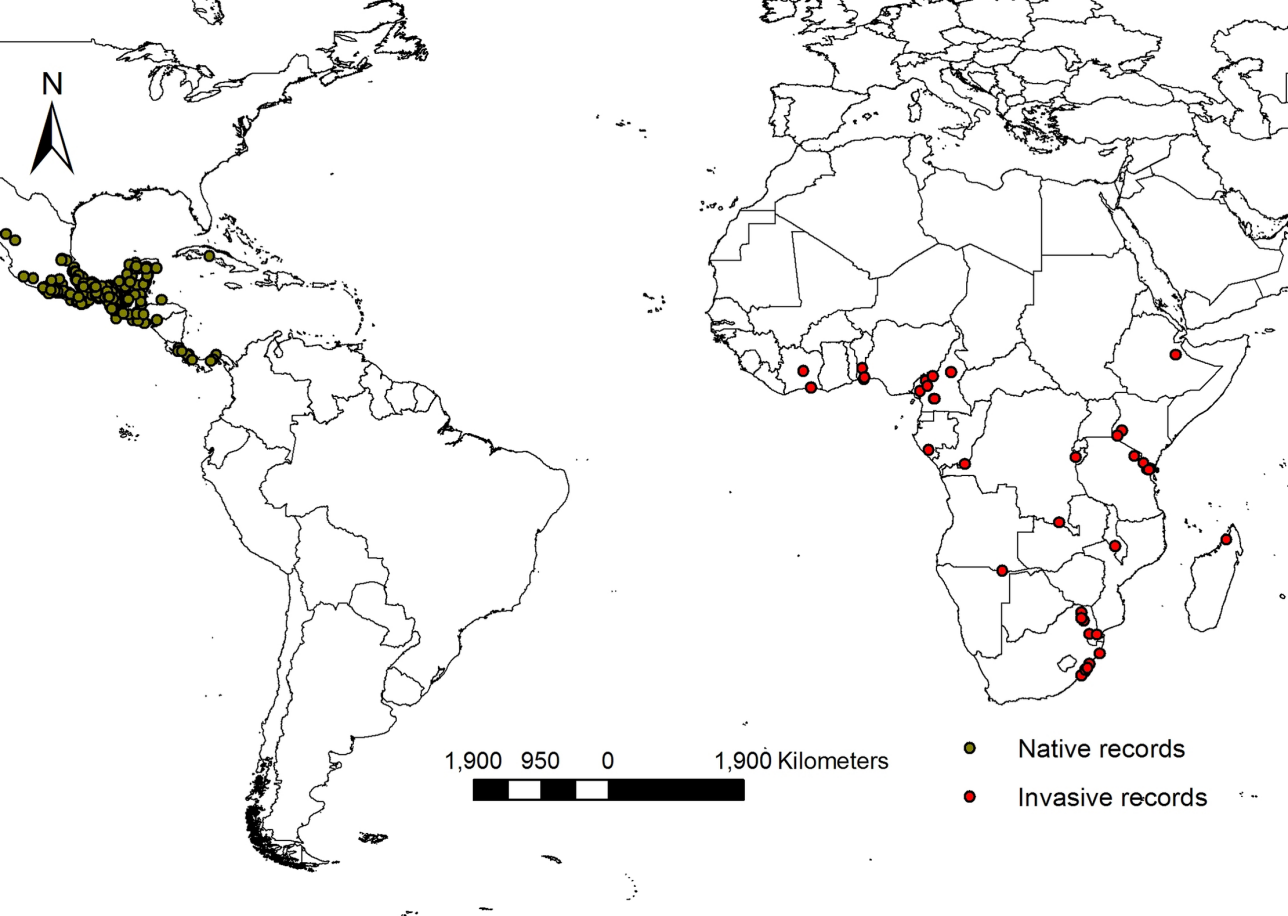
Geographic distribution of Mexican sunflower based on thinned occurrence records. Green and red circles represent native and introduced presences.

Niche dynamics analysis and distribution modelling involve the use of background points, i.e. points randomly sampled within a selected calibration area that represent the available environmental conditions. This area must represent the extent that could have been reasonably reached by the species given its dispersal ability. In the absence of detailed information regarding the natural dispersal distance of *T*. *diversifolia*, we defined Mexican sunflower’s native range as the entire Central America, between the longitudes 118.5° W and 77.5° W and between the latitudes 5.5° N and 32.7° N (Fig 1). In Africa, we have evidence that *T*. *diversifolia* has already spread throughout a large part of the continent via human transport. Therefore, we defined the species’ invasive range as the entire African continent. These native and invasive ranges will be used as calibration areas from which background points will be sampled in subsequent analyses.

### Climate data

Current (1960–2000) and future (2041–2060) climate data were downloaded from the WorldClim database [52,53] at a resolution of 2.5 arc-minutes. Data at this resolution generally provide better predictions for sessile organisms as plants [54]. The WorldClim database consists of 19 derived bioclimatic variables that represent climate average, extremes and variability, which are usually important in determining species distributions at a global scale [36,55]. Furthermore, just like many tropical invasive plants, detailed autecological information on *T*. *diversifolia* is not available thereby making it difficult to select *a priori* a specific set of biologically relevant predictors. Because collinearity between variables affects model performance [56], the variance inflation factor (VIF) was used to exclude correlated bioclimatic variables one by one using a stepwise method in the usdm R package [57]. Variables with a VIF greater than 10 indicate collinear data [58]. Using this method, we identified nine bioclimatic variables with a VIF between 2.3 and 5.7, which were selected for subsequent analyses (S1 Table). We selected two commonly used models to assess the future distribution of this species in Africa: the Hadley Global Environment Model (HadGEM2-ES) and the Model for Interdisciplinary Research on Climate (MIROC5). The projections were run under the extreme Representative Concentration Pathway (RCP) 8.5, which corresponds to high greenhouse gas emission rates [59]. This way, we evaluated the largest possible impact of climate change on *T*. *diversifolia*’s distribution, allowing us to forecast potentially invaded areas in the worst-case scenario.

### Niche analysis

We used the PCA-env approach of Broennimann [60] and Petitpierre [37] to measure the niche of *T*. *diversifolia* in relation to climatic factors present in its native and introduced ranges. We summarized the climatic space made up of the nine variables (S1 Table) on the two first axes of a Principal Component Analysis and divided this reduced space into a grid of 200 × 200 cells. Then we used a kernel density function to convert occurrences of *T*. *diversifolia* in each cell into 'smoothed' densities of occurrences. Similarly 10,000 points were randomly generated from each range and used to estimate densities of available environments. Niche overlap was estimated using Schoener’s D index [61], which ranges from 0 (no niche overlap) to 1 (complete niche overlap). To assess whether the native and invaded niches of this species are identical and similar, we performed tests of niche equivalency and similarity [61]. In these two tests, values of niche overlap, D were compared to those observed from the null distribution of 1000 random replicates [60] to test for significant niche conservatism between both ranges. Specifically, the niche equivalency test assessed whether the species’ realized niches in the native and introduced ranges were more equivalent than expected by chance, through a random permutation of the whole pool of occurrences. By contrast, the niche similarity test assessed whether the species occupied a niche in its introduced range that was significantly more similar than the one occupied in its native range, by randomizing occurrences within the invaded range only. Following the framework of Guisan [42], we calculated the 'unfilling' index *U*, which depicts the proportion of the climatic niche occupied by *T*. *diversifolia* in its native range exclusively, the 'expansion' index *E*, which represents the fraction of the invaded niche non-overlapping with the native niche and niche 'stability' (*S*), the climatic space filled by this species in both ranges. Analyses were performed in R [62] using the “ecospat” package [63].

### Reciprocal distribution modelling

To estimate the potential distribution of *T*. *diversifolia* in its invaded range, we used the Reciprocal Distribution Modelling approach as described in Fitzpatrick [64]. Briefly, a first model was calibrated on the native range (i.e., using native occurrences) and projected to the invaded range. Then, a second model was calibrated using occurrence data from the invaded range, in Africa and projected on the native range. Each model was also projected in the same area as its calibration occurrences. The degree of similarity between observed (calibrated in the same geographical extent) and projected models was assessed.

The potential distribution of *T*. *diversifolia* based on current climate (between 1960 and 2000) was generated using the Maximum Entropy modelling algorithm (MaxEnt) version 3.4.1 [65,66]. This method was chosen because of its widespread use and its proven performance for modelling species distribution with presence-only data [67–69]. Because MaxEnt's default regularization parameter and feature classes have profound impacts on model performance [70], we used ENMeval [71] to build a series of models with all possible combinations of these parameters. ENMeval produced a total of 48 models using a combination of six feature classes (based on linear (L), quadratic (Q), product (P), hinge (H) and threshold (T) responses to environmental gradients: L, H, LQ, LQH, LQHP, LQHPT) and eight regularization multipliers (0.5, 1.0, 1.5, 2.0, 2.5, 3.0, 3.5, 4.0). All models were built using 10,000 background points randomly selected within the calibration area (Central America for the model calibrated with native records and Africa for the model calibrated with invasive records).We used the “block” method implemented in ENMeval to partition data into four geographically distinct calibration and evaluation datasets, in order to conduct spatially independent tests of model performance [72,73] We selected models with a combination of feature class and regularization multiplier that provided the best trade-off between model goodness of fit and complexity using the Akaike information criterion corrected for small sample sizes (ΔAICc < 2) [74]. The predictive performance of these models was further assessed using the Boyce index [75], using default parameters of the "ecospat" package [63]. This index is a presence-only evaluation statistics that varies between -1 and + 1. Negative values of the Boyce index indicate that a model predicts low habitat suitability where a species actually occurs; values close to zero suggest a random model whereas positive values mean that the prediction of a model is consistent with observed presences [76]. We calculated the Boyce index in two ways: (i) using occurrences in the same area as model calibration to assess the interpolation performance of the model, and (ii) using model projection and occurrences in the other area to assess its extrapolation performance.

The complementary log-log output of MaxEnt was used to produce an estimate of occurrence probability in current and future climate as recommended by Phillips [77]. The minimum training presence and the 10^th^ percentile training presence threshold values were used to produce binary maps of suitable habitats for *T*. *diversifolia* in ArcGIS 10.5 (ESRI Inc.). We calculated from these binary maps the area of suitable habitat in current and future climates. Furthermore, we produced a map of climate suitability in the invasive range by merging native and invasive projections based on their maximum predicted value.

## Results

### Niche analysis

**Fig 2:**
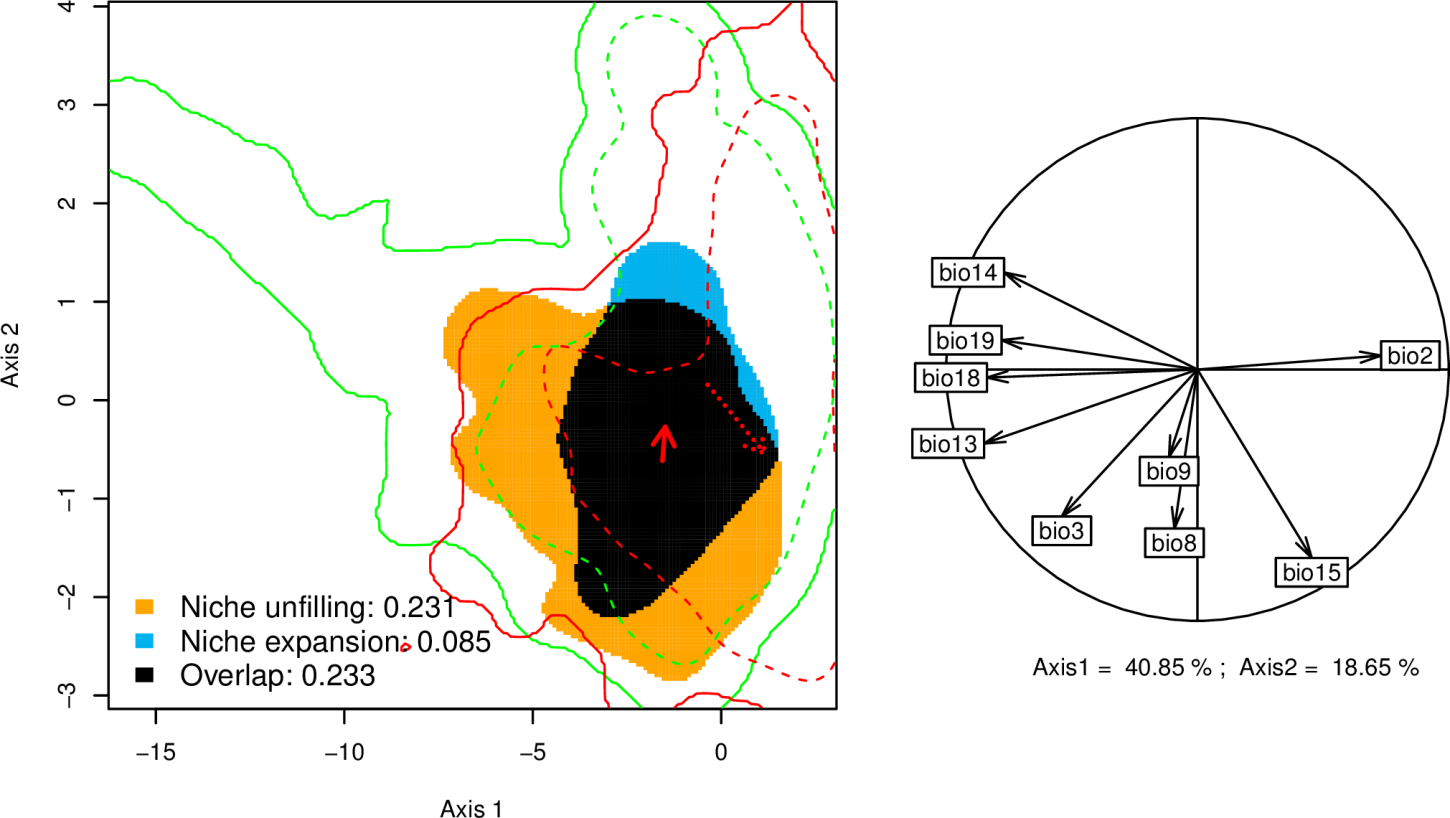
Principal component analysis of niche shift for *T*. *diversifolia*. The green and red contour lines demarcate the available niche in the native and invaded range respectively. The solid and dashed contour lines illustrate respectively 100% and 50% of the available environment. The correlation circle indicates the weight of each bioclimatic variable on the niche space defined by the first two principal component axes.

The climatic space occupied by *T*. *diversifolia* in its native and invaded ranges is represented in Fig 2. The correlation circle shows that the first two PCA axes explained 59.5% of the variance in the set of nine bioclimatic variables. Thermal variables were the most important variables to both principal components with the first and second axes positively associated with mean diurnal range (BIO 2) and isothermality (BIO 3) respectively (Table 1). Niche overlap of *T*. *diversifolia* between Central America and Africa was low (Schoener’s D = 0.232) following the classification scheme of Rödder and Engler [78]. The observed niche overlap was significantly more equivalent (*P* = 1) or more similar (*P =* 0.22) than D values obtained from a null distribution. This suggests that our species does not occupy similar niches in both ranges. Accordingly, the relatively high 'unfilling' index (*U* = 0.231) intimates that *T*. *diversifolia* occupies in its invaded range a small fraction of suitable climatic conditions. Nevertheless, the very high 'stability' index obtained here (*S* = 0.915) and the low 'expansion' index (*E* = 0.085) support the hypothesis of niche conservatism for this species in Africa.

**Table 1:** Loadings on two PCA axes of variables used to test climatic niche shift of *T*. *diversifolia*.

Climatic variable	Axis 1 (40.85%)	Axis 2 (18.65%)
**BIO 2**	1	-0.314
**BIO 3**	-0.314	1
**BIO 8**	-0.084	0.232
**BIO 9**	-0.293	0.108
**BIO 13**	-0.544	0.608
**BIO 14**	-0.436	0.116
**BIO 15**	0.3367	0.111
**BIO 18**	-0.497	0.450
**BIO 19**	-0.484	0.318

### Model performance

Six out of the 48 models calibrated on the native range showed a balance between goodness-of-fit and complexity (ΔAIC < 2) while only one invasive model satisfied this condition (S2 Table). The best models were based on a regularisation constant of 3 and 4 for the native and invasive models respectively with their corresponding Linear, Quadratic, Hinge and Product (LQHP) and LQH feature classes. All MaxEnt models of *T*. *diversifolia* calibrated and projected in the native range showed good predictive power with a Boyce index > 0.96. This value was lower (0.33–0.79) when projected in the invasive range. On the contrary, models calibrated in the invasive range performed only moderately well when projected in the same area (Boyce index = 0.72), but were very efficient when transferred in the native range (Boyce index = 0.98) (S2 Table).

### Current and future distribution of *T*. *diversifolia*

**Fig 3:**
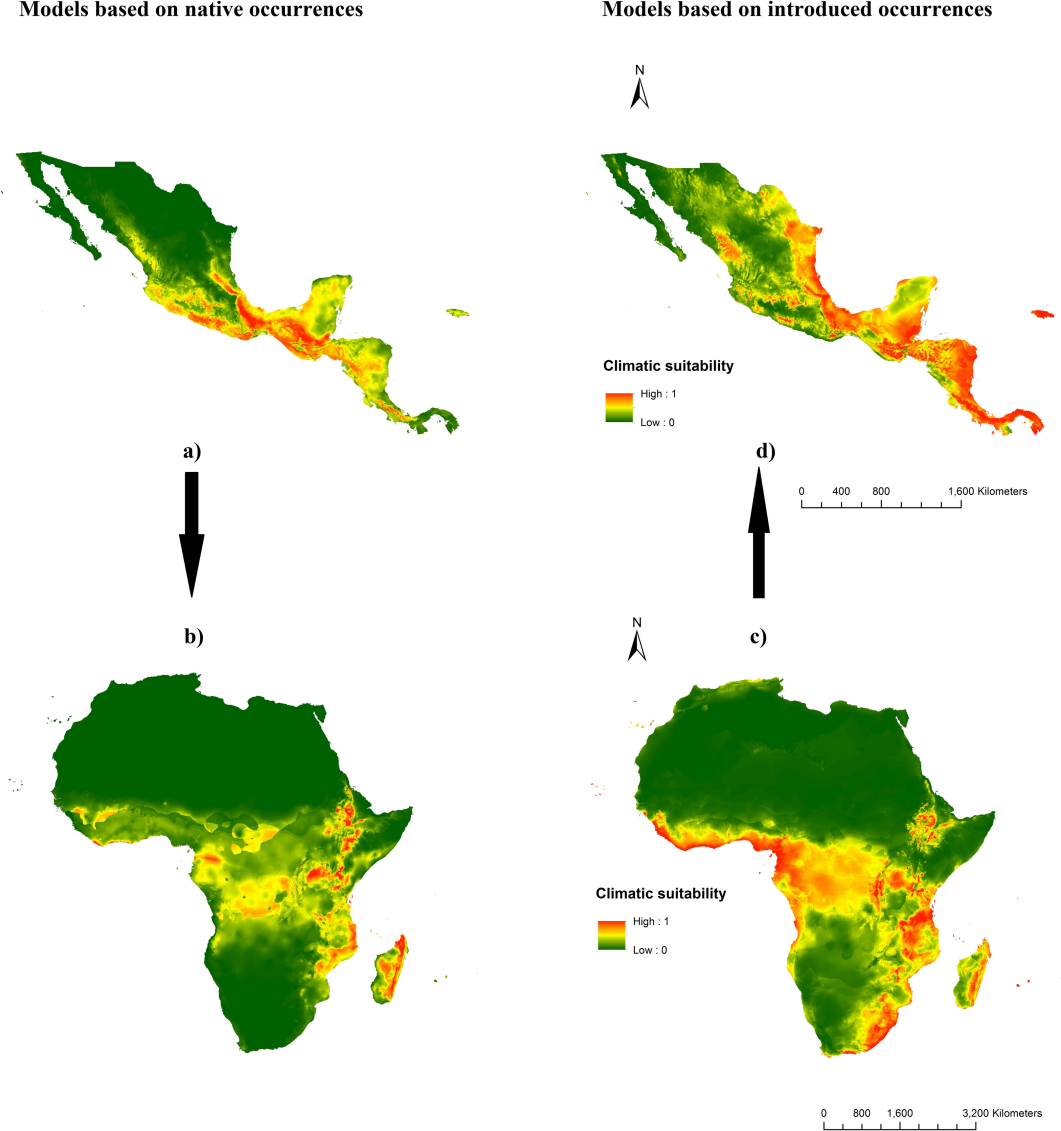
Potential distribution of *T*. *diversifolia* based on current (1960–2000) data. Native distribution (a) and projected distribution (b) based on 251 occurrences in Central America. Models calibrated in Africa with 39 records (c) and projected to the native range (d).

The potential distribution of *T*. *diversifolia* based on current climate and occurrence records (1960–2000) is shown in Fig 3. The model trained on the native range showed that most of northern Mexico and Panama are climatically unsuitable for this species (Fig 3A). Based on the minimum training presence threshold, 77% of the climatic space in the entire native range of *T*. *diversifolia* is suitable for this species (S1 Fig). This space was reduced to 32% based on the 10th percentile training presence threshold (S2 Fig, Table 2). In contrast, the prediction of the reciprocal model (calibrated with invasive occurrences and projected onto the native range) differed as there was a notable increase in areas of very high habitat suitability mainly across the eastern coast of Mexico and throughout Jamaica (Fig 3D). The southern part of the native range especially Belize, Guatemala, Costa Rica, Panama and Jamaica presented high climatic suitability for *T*. *diversifolia*. Based on the minimum and the 10th percentile training presence thresholds this model predicted a broader distribution on *T*. *diversifolia*, 84% and 55% of the native range respectively (Table 2).

**Table 2:** Predicted areas of climatically suitable habitats for *T*. *diversifolia*. Suitable area (in Km^2^ with percentage of total area in brackets) is presented for models based on native and invasive records and projected in the native and invasive range. In addition, predicted suitable area in the invasive range is shown based on merged models, for current and future climate (2041–2060, RCP8.5) according to two global circulation models.

SN	Model	Predicted suitable area (Km^2^)	
		Minimum training presence	10th Percentile training presence
**1**	**Native → Native**	2,404,475 (77.05%)	1,012,550 (32.45%)
**2**	**Invasive → Native**	2,636,350 (84.48%)	1,730,875 (55.47%)
**3**	**Invasive → Invasive**	17,536,375 (47.59%)	10,590,825 (28.74%)
**4**	**Native → Invasive**	20,651,800 (56.05%)	4,338,475 (11.77%)
**5**	**Invasive → merged**	18,926,854 (63.36%)	17,672,097 (31.77%)
**6**	**Invasive → HadGEM2-ES**	18,632,599 (62.37%)	7,455,817 (24.96%)
**7**	**Invasive → MIROC 5**	18,985,624 (63.56%)	7,852,171 (26.29%)

The arrow indicates the range on which projection was made.

**Fig 4:**
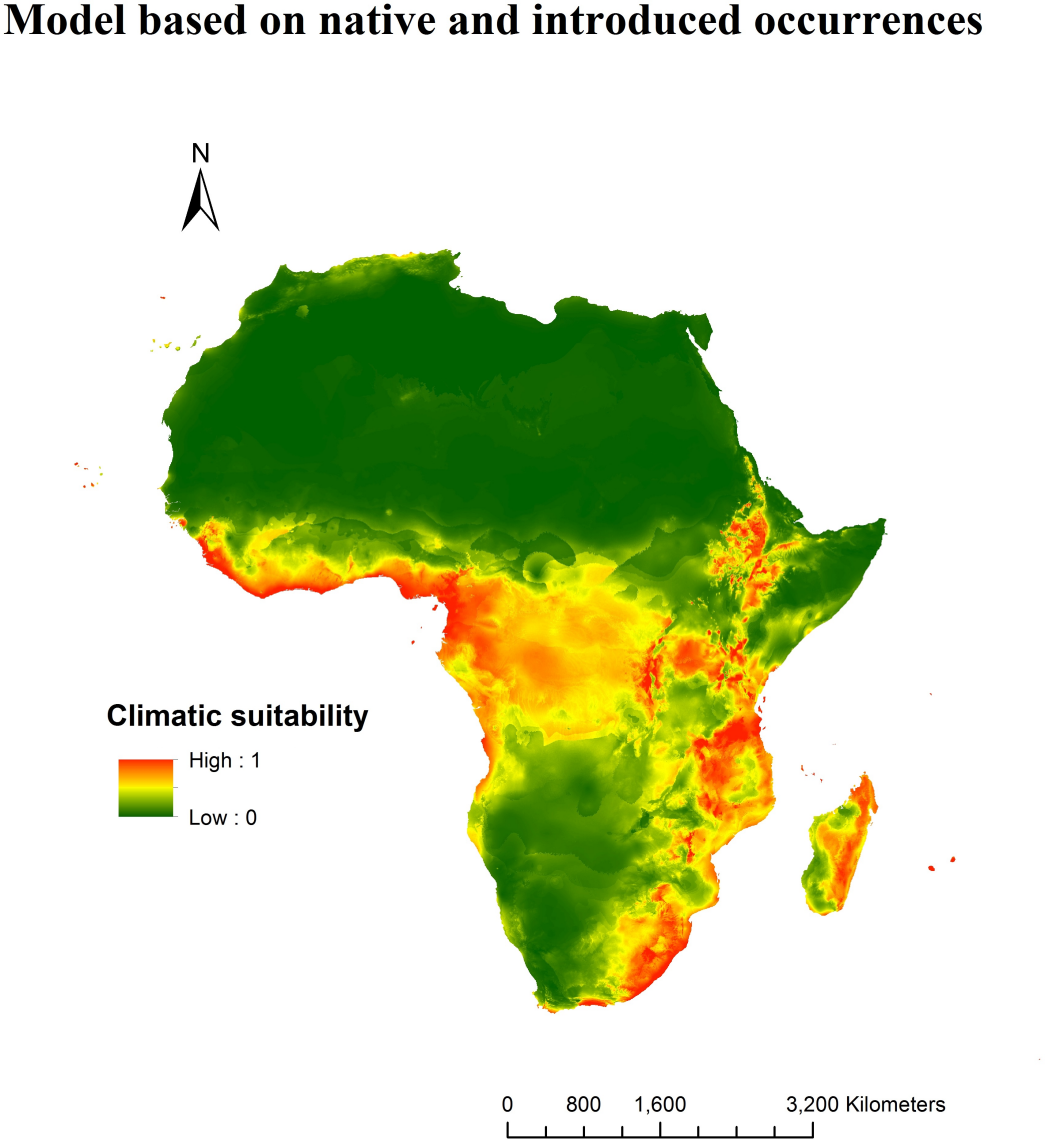
Current potential distribution of *T*. *diversifolia* based on presence records from both introduced and native ranges. Native and invasive projections were merged based on the maximum predicted value.

The MaxEnt model based on introduced presence records of *T*. *diversifolia* in Africa predicted high suitability in several regions of the African climatic space, mostly in East and West Africa and eastern Madagascar. These areas encompass much of Ethiopia, Kenya, Uganda, Rwanda, Burundi, Tanzania, Malawi, Congo, Gabon, Equatorial Guinea, Cameroon, Lesotho, Swaziland, South Africa and span from the Gulf of Guinea to Côte d'Ivoire along the Atlantic coast (Fig 3C). The current potential distribution of *T*. *diversifolia* in Africa based on native records generally detected similar regions with high climatic suitability as the reciprocal distribution model, based on introduced records (Fig 3B) Both predictions for Eastern Africa and Madagascar were consistent however, most of Western and Southern Africa were different. In effect, upon projection to Africa, the native model presented a low transferability as it noticeably failed to account for the presence of *T*. *diversifolia* in the whole of South Africa and many parts of West Africa (Fig 3B). However, the prediction of this model was consistent with that of its reciprocal especially for Madagascar. A combined prediction of both models based on the maximum predicted values was therefore used as a better representation the current distribution of this species in Africa (Fig 4). Based on the selected thresholds, much of the sub-Saharan climate was found to support the growth of this species (S1 and S2 Figs).

**Fig 5:**
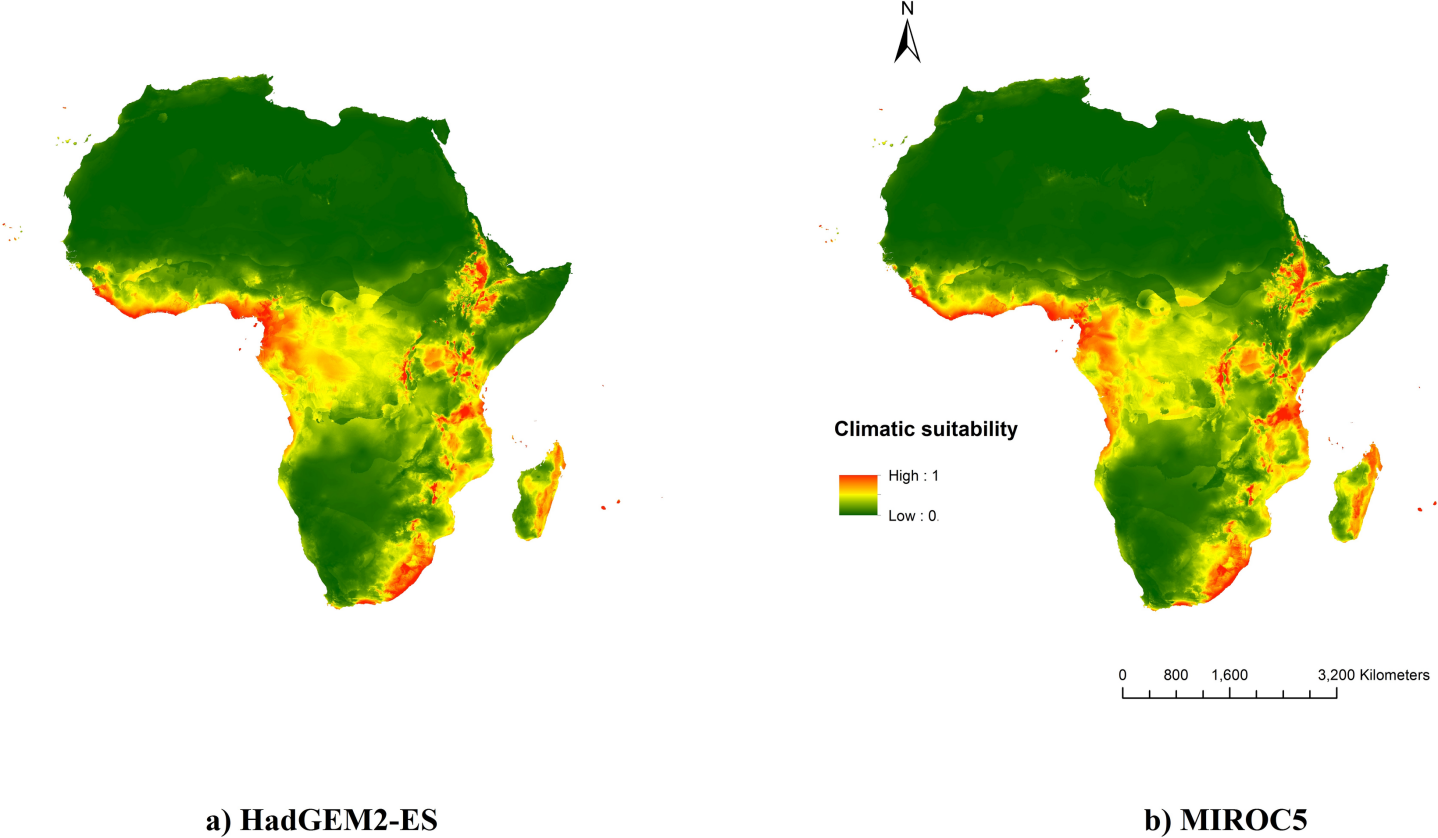
Future potential distribution of *T*. *diversifolia* based on 2041–2060 climate data. In all cases, the native and invasive projections were merged based on the maximum predicted value.

The potential distribution of *T*. *diversifolia* in Africa modelled with future climatic conditions followed the same spatial pattern as the current distribution. Surprisingly, even under the more extreme RCP 8.5 climate projections, climatic suitability was consistently reduced for both HadGEM2-ES and MIROC5 (Fig 5). It did not result in a diminution of suitable area according to the minimum training presence threshold (Table 2, S2 Fig). However, it corresponded to a reduction of ca. 57% of the suitable area when considering the 10^th^ percentile threshold (Table 2, S2 Fig).

### Climatic variable importance and response

The relative contributions of bioclimatic variables to the MaxEnt models showed that only three of these variables accounted for more than 80 percent in each model (Table 3). The four most significant variables affecting the distribution of *T*. *diversifolia* in its native range were in order of decreasing magnitude, precipitation of the wettest month (BIO 13), isothermality (BIO 3), mean diurnal range (BIO 2) and precipitation seasonality (BIO 15) (Table 3). Here, the total contribution of these variables was 92.4% (Table 3). In the invaded range, the distribution of this species was constrained by precipitation of the warmest quarter (BIO 18), precipitation of driest month (BIO 14) and mean diurnal range (BIO 2), which together explained 93.3% of its distribution.

**Table 3:** Percent contribution and permutation importance of the selected bioclimatic variables in MaxEnt models for *T*. *diversifolia*.

	Native model		Invasive model	
Variables	% Contribution	Permutation importance	% Contribution	Permutation importance
**bio 2**	8.74	8.34	13.64	30.1
**bio 3**	24.80	22.50	0.26	0.00
**bio 8**	0.63	2.37	0.00	0.00
**bio 9**	0.79	8.06	5.10	30.30
**bio 13**	51.36	10.54	1.26	6.78
**bio 14**	3.29	21.77	22.27	9.70
**bio 15**	7.54	18.58	0.00	0.07
**bio 18**	0.05	0.04	57.42	20.89
**bio 19**	2.80	7.78	0.05	1.27

**Fig 6:**
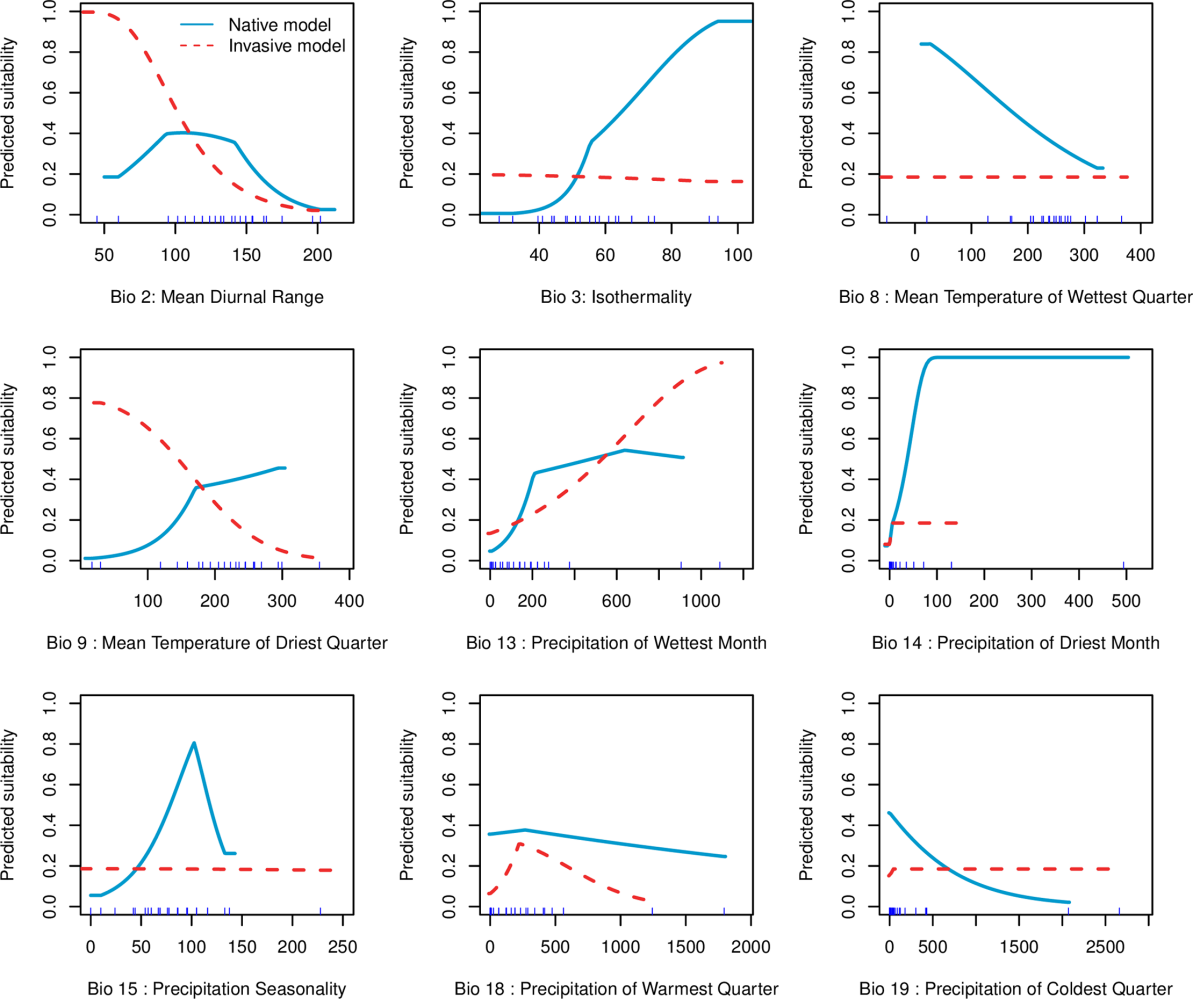
Response curves of the nine uncorrelated climatic variables used in modelling the distribution of *T*. *diversifolia* in Africa and Central America. Response curves in blue and red lines represent the native and invasive models respectively.

In the native range, the predicted climatic suitability for *T*. *diversifolia* was positively correlated with precipitation of the wettest month (BIO 13), isothermality (BIO 3) and precipitation of the driest month (BIO 14) suggesting that these variables may be effective predictors for modelling the potential distribution of this species here (Fig 6). For isothermality values of approximately 10^o^ C, predicted habitat suitability was near 40%. This decreased to 0 as isothermality exceeded 20^o^ C. When precipitation seasonality (BIO 15) fell below 0.1, predicted suitability for this plant was less than 10%. When BIO 15 varied from 0.1 to ≈ 1, while others variables were kept at their mean value, suitability increased to 80%. Afterwards, an abrupt decrease was recorded followed by a plateau from 1.3. Predicted climatic suitability of *T*. *diversifolia* in Africa was relatively low (<40%) for the two most important variables (BIO 18 and BIO 14) when all others were held at their average value. Mean diurnal range (BIO 2) and precipitation of the driest month (BIO 9) were positively correlated with predicted suitability, which dropped to less than 5% when BIO 2 and BIO 9 were above 20^o^ C and 35^o^ C respectively (Fig 6).

## Discussion

There is no consensus on the incidence of climatic niche conservatism or shift in invasive terrestrial plants [37,39,40,41]. In agreement with Petitpierre [37] and Atwater [41], our results highlight climatic niche conservatism in *T*. *diversifolia*. We showed that populations of this plant in Africa occupy a subset of its native Central American climatic niche, with limited niche expansion. A similar finding was reported for the cosmopolitan *Lantana camara*, which also presented a large extent of niche unfilling in its invaded range in Africa [79].

The exact time span since the introduction of Mexican sunflower is unknown is many African countries. The only available record indicates less than 50 years have passed since its introduction in Nigeria [14]. This short time span likely explains why we observed very little niche expansion in the invaded range. Niche conservatism is usually frequent over short time periods mainly because of slow evolutionary changes [33]. Thus, at the geographical extent at which this study was limited, *T*. *diversifolia* presents another case of niche conservatism in line with the findings of Petitpierre [37]. As pointed out by Goncalves [79], further analyses outside Africa may reveal a niche shift. In China for example, this species has a relatively longer and richer invasion history [80,81] that may have allowed it to adapt to different climates. Unfortunately, it was not possible to include regions outside the defined study areas in our analyses as the number of relevant occurrence data was unexpectedly low.

Although Mexican sunflower is widespread in its native range, our study has shown that this species does not occupy all native areas with suitable climatic conditions, therefore suggesting a geographic range unfilling, which can affect the results of niche assessments [42]. The capacity of this plant to invade new regions can be further appreciated by its ability to hybridize with closely related "native invasive'' species including *T*. *rotundifolia* and *T*. *tubaeformis* [82,83]. Moreover, over longer time periods, and depending on the actions that are initiated to halt the spread of Mexican sunflower, this plant may continue its expansion in Africa and eventually fill its full climatic niche.

Our niche models showed a good overall performance in both ranges suggesting their ability to discriminate between occurrence and absence areas for *T*. *diversifolia*. The model calibrated on the native range and projected to Africa showed good transferability as it could successfully predict most of the test points. Although we used an approach adequate for studies that require transferring presence-only models across space [71], this model was undermined by prediction mismatches in some parts of Western and Southern Africa where the focal species has been recorded. This outcome seems rather frequent for invasive species [84]. Fernández and Hamilton [84] showed that climatic niche models of the invasive plants *Lantana camara*, *Mimosa pigra* and *Leucaena leucocephala* are almost completely transferable to invaded ranges whereas other invaders' niches show low to very low transferability in space. Spatiotemporal transferability of ecological models is vital for accurately predicting biological invasions [85]. Proximality, the use of ecologically relevant predictors is one of the factors that impinge on the transferability of species distribution models [86]. Our models may suffer from low proximality as the real physiological requirements of *T*. *diversifolia* could not be identified in the literature. Transferability is also improved by pooling occurrence record from introduced and native regions [35,87] and carrying out density-based occurrence thinning [88]. Spatial bias could not have affected transferability here as occurrence records within a distance of 5 km were removed using spThin [49]. We therefore used reciprocal predictions between the native and invaded ranges of *T*. *diversifolia* as an optimal representation of its current distribution in Africa [64]. The prediction of the native model is in agreement with the observation of Lentz [89] who reported that *T*. *diversifolia* is at a disadvantage in the north of Mexico unlike in the south where it is unfettered by low temperatures and day length. Considering the relatively high unfilling index obtained, it is likely that this species has not yet attained its distribution equilibrium in its invaded range. This may explain this model's inability to detect areas where this species is currently present, especially in South Africa. The high climatic suitability of this species in Madagascar as given by our reciprocal models portends negative implications for the flora of this country.

Assessments of range dynamics suggest that species generally respond to climate change by shifting their distribution towards higher altitudes and latitudes [90]. Here, in contrast, our species distribution models did not predict such pattern but point to a relatively stable distribution of climatically suitable habitats even under the most severe climate change scenario. This has important consequences in terms of management perspectives. First, it suggests that the areas currently invaded will remain suitable for *T*. *diversifolia*, so that one may not expect this species to be naturally extirpated by climate change, except perhaps in a few restricted areas. Second, it also makes possible to implement management actions on specific areas of potential invasion, knowing that they will not change over time. Actively monitoring the distribution of *T*. *diversifolia* both in its native and introduced ranges may provide more insights into its range dynamics under climate change. So far, empirical estimates of climate-driven range shifts have mainly involved animal taxa while terrestrial plants remain less understood so that their actual response to climate change is uncertain [91].

Our analysis of the niche of *T*. *diversifolia* revealed that this species is limited to the more humid regions of Africa. This result is corroborated by our small-scale observation in South Western Nigeria where *T*. *diversifolia* completes its growth cycle just before the dry season (December-March), which corresponds to the driest quarter of the year. The initial set of 19 bioclimatic variables was reduced to three useful ones as opposed to several related studies (e.g. [92,93]) where at least four bioclimatic variables were found important. Although mean temperature of the wettest quarter showed little change between the two ranges, the slight difference in the other most important variables found here is similar to that reported by Suárez-Mota [92] for reciprocal models of the invasive *Chromolaena odorata* in America and Africa. Similar trends were reported by Hernrandez-Lambraño [93] suggesting that predictor variables contributing to a model calibrated in one region may change upon projection to another.

This study showed that optimal MaxEnt parameters for the same species vary according to the calibration area/environment and occurrence data thereby highlighting the importance of specifically tuning these parameters [94]. The failure of the invasive model to capture some parts especially South Africa confirms that the use of presence records from both introduced and native ranges improves prediction of niche models [95].

In conclusion, our results predicted that large areas, mainly in Sub-Saharan Africa are climatically suitable for *T*. *diversifolia*. Although with changing conditions, especially disturbance regimes, which were not considered in this study [96], increasing deforestation and agricultural expansion may favour the spread of this species in the predicted climatically suitable areas. Thus, a timely intervention may be necessary to prevent further invasions by this species.

## Supporting information

S1 Table List of bioclimatic variables used for this study. (DOCX)

S2 Table Performance metrics of models produced using ENMeval and Boyce index. (DOCX)

S1 Fig Binary predictions of current potential distribution of *T*. *diversifolia* in its invaded range based on merged native and invasive models. (TIF)

S2 Fig Binary predictions of future potential distribution of *T*. *diversifolia* in its invaded range. (TIF)
