# Expression of Concern: Climatic niche and potential distribution of *Tithonia diversifolia* (Hemsl.) A. Gray in Africa

**DOI:** 10.1371/journal.pone.0358418

**Published:** 2026-09-17

**Authors:** The PLOS One Editors

The *PLOS One* Editors issue this Expression of Concern because this article [1] was identified as one of a series of submissions for which we have concerns about the integrity and/or reliability of the peer review process.

Readers are advised to interpret the article [1] with consideration of this issue. In following up on this matter, PLOS did not evaluate the article’s integrity or scientific validity.

The Editors do not have concerns regarding the authors’ conduct during the peer review process.
