# Supplementary material for: Climatic niche and potential distribution of Tithonia diversifolia (Hemsl.) A. Gray in Africa
Source: PLoS One. 2018 Sep 5;13(9):e0202421. doi: 10.1371/journal.pone.0202421 (PMC6124709; doi:10.1371/journal.pone.0202421)
Supplement: S1 Table — (DOCX) [file pone.0202421.s001.DOCX]

**S1 Table. Correlation between selected bioclimatic variables.** (A) List of bioclimatic variables with a Variance Inflation Factor (VIF) < 10, (B) Pairwise Pearson correlations between variables in the native and invasive ranges

(A)

| **Bioclimatic variable** | **Label** | **VIF** |
| --- | --- | --- |
| Mean Diurnal Range (Mean of monthly (max temp - min temp)) | bio2 | 2.32 |
| Isothermality (BIO2/BIO7) | bio3 | 4.41 |
| Mean Temperature of Wettest Quarter | bio8 | 2.35 |
| Mean Temperature of Driest Quarter | bio9 | 3.46 |
| Precipitation of Wettest Month | bio13 | 5.70 |
| Precipitation of Driest Month | bio14 | 3.38 |
| Precipitation Seasonality ( Coefficient of Variation ) | bio15 | 2.35 |
| Precipitation of Warmest Quarter | bio18 | 4.17 |
| Precipitation of Coldest Quarter | bio19 | 3.46 |

(B)

|  | **BIO2** | **BIO3** | **BIO8** | **BIO9** | **BIO13** | **BIO14** | **BIO15** | **BIO18** | **BIO19** |
| --- | --- | --- | --- | --- | --- | --- | --- | --- | --- |
| **BIO2** | -- | -0.416 | -0.271 | -0.563 | -0.673 | -0.631 | 0.541 | -0.543 | -0.608 |
| **BIO3** | -0.496 | -- | -0.173 | 0.430 | 0.622 | 0.308 | -0.073 | 0.417 | 0.439 |
| **BIO8** | 0.123 | 0.142 | -- | 0.593 | 0.126 | 0.141 | -0.053 | 0.105 | 0.122 |
| **BIO9** | -0.169 | -0.011 | 0.079 | -- | 0.498 | 0.344 | -0.185 | 0.302 | 0.438 |
| **BIO13** | -0.520 | 0.650 | 0.021 | 0.017 | -- | 0.628 | -0.166 | 0.799 | 0.671 |
| **BIO14** | -0.445 | 0.530 | -0.058 | 0.020 | 0.326 | -- | -0.643 | 0.640 | 0.681 |
| **BIO15** | 0.290 | -0.084 | 0.551 | 0.072 | 0.001 | -0.406 | -- | -0.149 | -0.484 |
| **BIO18** | -0.557 | 0.629 | -0.026 | -0.203 | 0.714 | 0.523 | -0.220 | -- | 0.470 |
| **BIO19** | -0.418 | 0.431 | -0.066 | 0.211 | 0.531 | 0.418 | -0.240 | 0.253 | -- |

Above diagonal: native range

Below diagonal: African invasive range
