# Supplementary material for: Climatic niche and potential distribution of Tithonia diversifolia (Hemsl.) A. Gray in Africa
Source: PLoS One. 2018 Sep 5;13(9):e0202421. doi: 10.1371/journal.pone.0202421 (PMC6124709; doi:10.1371/journal.pone.0202421)
Supplement: S2 Table — (DOCX) [file pone.0202421.s002.DOCX]

**S2 Table: Metrics produced by tuning with ENMeval.**

| Models | Feature Class | RM | Mean AUC | Mean AUC_DIFF_ | Mean OR_MTP_ | Δ AICc | Boyce index^1^ | Boyce index^2^ |
| --- | --- | --- | --- | --- | --- | --- | --- | --- |
|  | LQHP | 3.0 | 0.85 | 0.04 | 0.00 | 0.00 | 0.981 | 0.791 |
|  | LQHPT | 3.0 | 0.85 | 0.04 | 0.00 | 0.00 | 0.981 | 0.774 |
| Native | LQHP | 2.0 | 0.85 | 0.04 | 0.00 | 0.08 | 0.965 | 0.654 |
|  | LQHPT | 2.0 | 0.85 | 0.04 | 0.00 | 0.08 | 0.965 | 0.654 |
|  | LQHP | 1.5 | 0.86 | 0.04 | 0.00 | 0.26 | 0.988 | 0.329 |
|  | LQHPT | 1.5 | 0.86 | 0.04 | 0.00 | 0.26 | 0.988 | 0.329 |
| Invasive | LQH | 4.0 | 0.89 | 0.04 | 0.06 | 0.00 | 0.723 | 0.981 |

Six out of the 48 models calibrated on the native range showed a balance between goodness-of-fit and complexity (Δ AICc < 2). MaxEnt settings: RM (regularization multiplier) and feature classes (Linear L, Quadratic Q, Hinge H, Product P and Threshold T).

^1^Boyce index calculated based on occurrences in the calibration area

^2^Boyce index calculated based on model projection and occurrences in the other area (Africa for the native model and Central America for the invasive model)
