# Supplementary figures and images for: Climatic niche and potential distribution of Tithonia diversifolia (Hemsl.) A. Gray in Africa
Source: PLoS One. 2018 Sep 5;13(9):e0202421. doi: 10.1371/journal.pone.0202421 (PMC6124709; doi:10.1371/journal.pone.0202421)

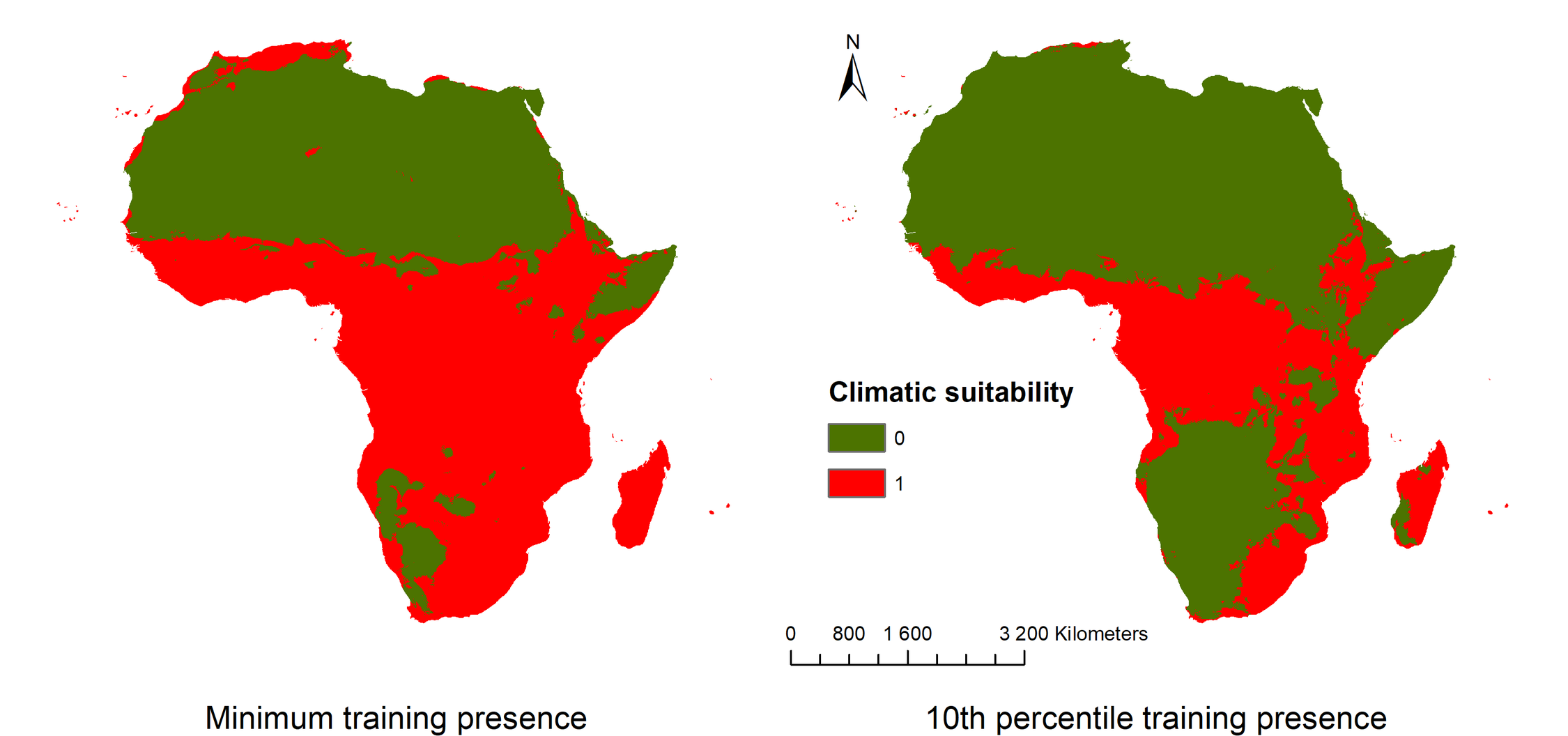

Supplement: S1 Fig — (TIF) [file pone.0202421.s003.tif]

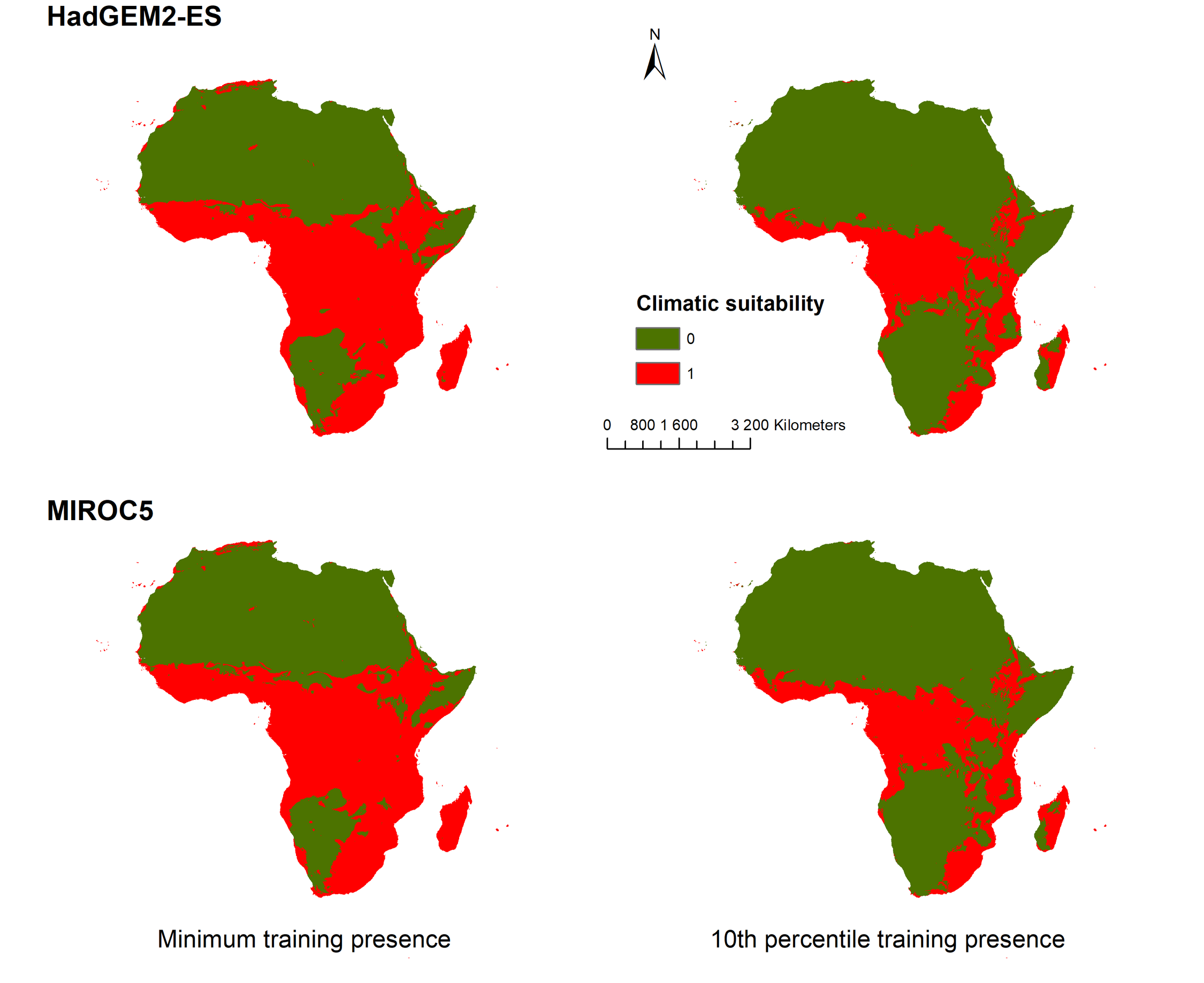

Supplement: S2 Fig — (TIF) [file pone.0202421.s004.tif]
